# Brains from non-Alzheimer’s individuals containing amyloid deposits accelerate Aβ deposition *in vivo*

**DOI:** 10.1186/2051-5960-1-76

**Published:** 2013-11-18

**Authors:** Claudia Duran-Aniotz, Rodrigo Morales, Ines Moreno-Gonzalez, Ping Ping Hu, Claudio Soto

**Affiliations:** Mitchell Center for Alzheimer’s Disease and Related Brain Disorders, Department of Neurology, University of Texas Houston Medical School, Houston, TX 77030 USA; Facultad de Medicina, Universidad de los Andes, Av. San Carlos de Apoquindo 2200, Las Condes Santiago, Chile; Education Ministry Key Laboratory on Luminescence and Real-Time Analysis, College of Life Sciences, Southwest University, Chongqing, China; Laboratory of Cellular Stress and Biomedicine, Institute of Biomedical Sciences, School of Medicine, University of Chile, 1027 Independencia, PO Box 70086, Santiago, Chile

## Abstract

**Background:**

One of the main features of Alzheimer’s disease (AD) is the presence of Aβ deposits, which accumulate in the brain years before the onset of symptoms. We and others have demonstrated that cerebral Aβ-amyloidosis can be induced *in vivo* by administration of AD-brain extracts into transgenic mice. However, it is currently unknown whether amyloid formation can be induced using extracts from individuals harboring Aβ deposits, but not clinical disease.

**Results:**

In this study we analyzed the amyloid-inducing capability of samples from individuals affected by mild cognitive impairment (MCI) and Non-Demented persons with Alzheimer’s disease Neuropathology (NDAN). Our results show that inoculation of transgenic mice with MCI and NDAN brain samples accelerated Aβ pathology in a similar way as extracts from confirmed AD.

**Conclusions:**

This data demonstrate that the sole presence of Aβ aggregates in a given sample, regardless of the clinical condition, is capable to accelerate Aβ deposition *in vivo*. These findings indicate that the amyloid-inducing activity may be present in the brain of people during pre-symptomatic or a-symptomatic stages of AD.

## Background

Alzheimer’s disease (AD) is a prevalent brain disorder, mostly affecting individuals over 65 years old [1, 2]. Clinically, this progressive and irreversible neurodegenerative illness is characterized by cognitive decline, which invariably leads to dementia [2]. The hallmark neuropathological lesions of AD brain are the extracellular deposition of misfolded amyloid-β (Aβ) as amyloid plaques and the formation of neurofibrillary tangles composed by intracellular aggregates of hyper-phosphorylated tau [3]. Aβ aggregates in AD can be found in a variety of arrangements such as soluble Aβ oligomers, diffuse deposits, dense core senile plaques, vascular deposits, and intra-cellular aggregates, among others [3, 4]. To different degree, all these structures have been associated to cell toxicity and tissue dysfunction [5–9].

Accumulation of Aβ aggregates in the brain is thought to begin many years or even decades before the onset of AD clinical symptoms [10–13]. Indeed, abundant amounts of Aβ deposits are detected in the brain of some subjects affected by mild cognitive impairment (MCI) [14, 15], which is considered a precursor stage of AD [16, 17]. Most patients with amnestic MCI do not meet the full neuropathologic criteria for AD, but their pathological features suggest a transitional state evolving towards AD [14, 18]. MCI is a clinical condition usually defined by subtle memory changes that do not significantly affect daily life [19]. MCI does not meet diagnostic criteria for dementia; however, people affected by this condition are at high risk to convert into AD [16, 19, 20]. Alternatively, it is widely known that elderly people usually exhibit cerebral Aβ pathology, even without any signs of dementia [11, 12, 21]. In fact, it is not uncommon to find cases of aged non-demented subjects harboring abundant amyloid lesions in their brains (here referred as Non-Demented individuals with Alzheimer’s disease Neuropathology or NDAN). These cases suggest that certain arrangements of misfolded Aβ are not associated to a clinical phenotype or that some people can cope with accumulation of misfolded aggregates [11, 21].

Recent studies have demonstrated that inoculation of AD brain homogenates is able to accelerate amyloid deposition in AD-transgenic mice [22, 23]. Furthermore, AD samples are also able to induce *de novo* Aβ pathology in animal models that do not spontaneously develop this type of lesions during their whole lifespan [24, 25]. These findings suggest that the accumulation of Aβ deposits follows a seeding-dependent process of misfolding and aggregation that can be induced in a prion-like manner by administration of preformed aggregates [26–30]. Experimental induction of amyloid pathology has been achieved by injection of brain samples from AD patients [22–25] and aged AD-transgenic mice [23, 31, 32]. However, there are no reported studies investigating whether the pathological induction can also be observed upon inoculation of brain samples from persons at potentially pre-symptomatic or a-symptomatic stages of AD, which contain substantial cerebral amyloid deposits, but not overt dementia. In the present study, we evaluated the Aβ seeding capability of MCI and NDAN brains in AD-transgenic animals. Strikingly, we found that MCI and NDAN samples can exacerbate amyloid deposition to a similar or even greater extent than AD specimens.

## Methods

### Human samples

AD (79 years old, female, AD clinical diagnosis that was confirmed post-mortem), NDAN (81 years old, male, non-demented diagnosis) and aged control (59 years old, male, non-demented diagnosis) brain samples were obtained from the National Disease Research Interchange (Philadelphia, PA, USA). MCI (95 years old, male, MCI diagnosis by neuropsychological test) sample was kindly provided by Dr. Eliezer Masliah (University of California at San Diego, California, USA). All areas analyzed corresponded to the cingulate cortex. Research on human samples was performed following The Code of Ethics of the World Medical Association (Declaration of Helsinki). Informed consent was obtained for experimentation with human subjects. Samples were manipulated following the universal precautions for working with human samples and as directed by the Institutional Review Board of The University of Texas Medical School at Houston.

### Transgenic mice

APP_Swe_/PSEN1_∆E9_ transgenic mice were obtained from Jackson Laboratory (Bar Harbor, ME, USA). These mice over-express the human version of amyloid precursor protein (APP) harboring the Swedish double mutation (K670M and N671L) and the human presenilin-1 protein with the DeltaE9 mutation (PSEN1-∆E9) [33]. Treated animals were housed in groups of up to 5 in individually ventilated cages under standard conditions (22°C, 12 h light–dark cycle) receiving food and water *ad libitum*. All animal manipulation was in agreement with NIH guidelines and approved by the Animal Welfare Committee of the University of Texas – Medical School at Houston. 5–8 animals per experimental group were used as indicated in each section. Males and females were indistinctly used (overall 43% males, 57% females).

### Preparation and characterization of human brain inocula

Frozen cingulate cortex sections of AD, MCI, NDAN and aged non-demented individuals were homogenized at 10% (w/v) in ice-cold PBS containing a cocktail of protease inhibitors (Roche Diagnostics GmbH, Mannheim, Germany). Resulting homogenates were stored at −80°C until used for animal injection. In order to measure the amount of insoluble Aβ in each case, 200 μL aliquots of each sample were centrifuged at 100,000×g for 1 h and 4°C using a L100K Beckman-Coulter ultracentrifuge (Beckman-Coulter, Brea, CA, USA). Supernatants were recovered and saved as “PBS fractions” and pellets were resuspended in 200 μL of a 2% SDS solution by pipetting and sonication. Samples were centrifuged as explained above and the supernatants were diluted 40 times in EC buffer (0.02 M phosphate buffer, pH 7, 0.4 M NaCl, 2 mM bovine serum albumin, 0.05% CHAPS and 0.05% sodium azide). Pellets were resuspended in 200 μL of 70% formic acid (FA) and centrifuged for 30 minutes using the same temperature and speed previously described. Resulting supernatants ("FA fractions") were diluted 20 times in 1 M Tris buffer (pH 11) to adjust pH. All resulting samples were stored at −80°C until measured by an ELISA kit able to specifically detect Aβ_40_ and Aβ_42_ (Invitrogen, Carlsbad, CA, USA). Histological characterization was performed as described below.

### Intra-cerebral inoculation of brain extracts

~30 days-old APP_Swe_/PSEN1_∆E9_ mice were intra-cerebrally injected with 10 μL of 10% (w/v) human cingulate cortex homogenate. Briefly, mice were anesthetized using isofluorane. Skin was incised and a small hole was drilled in the skull. Samples (brain homogenates and PBS) were injected into the right hippocampus using the following coordinates as measured from bregma: antero-posterior (AP), -1.8 mm; medio-lateral (ML), -1.8 mm; dorso-ventral (DV), -1.8 mm. When finishing treatment, skin was closed using surgical suture. Animals were placed on a thermal pad until recovery and monitored daily for several days. Mice were sacrificed by CO_2_ inhalation at ~150 days after treatment. Brains were removed and the right (injected) hemisphere was stored in 10% formalin for histological studies.

### Histological studies

10-μm-thick serial slices from all animal groups (n = 5-8/group; 5 sections/stain/animal) were processed in parallel for histological analyses. For immunohistochemistry, sections were deparaffinazed and the endogenous peroxidase activity was blocked with 3% H_2_O_2_/10% methanol in PBS for 20 min. Then, brain sections were incubated in formic acid 85% for 5 min for antigen retrieval. The sections were incubated overnight at room temperature with the mouse anti-Aβ antibody at a 1:1000 dilution (Covance, Princeton, NJ). After washing, sections were incubated for 1 h with an HRP-linked secondary sheep anti-mouse antibody at a 1:1000 dilution (GE Healthcare, Little Chalfont, UK). Peroxidase reaction was visualized using DAB Kit (Vector) following the manufacturer’s instructions. Finally, sections were dehydrated in graded ethanol, cleared in xylene, and cover-slipped with DPX mounting medium (Innogenex, San Ramon, CA). A similar approach was used for the detection of hyper-phosphorylated tau in human samples (AT8 antibody, 1:100 dilution; Pierce, Rockford, IL, USA). For ThS staining, sections were incubated in ThS (Sigma) solution (0.025% in 50% ethanol) for 5–10 min after deparaffinization. Sections were dehydrated in graded ethanol, cleared in xylene, and cover-slipped with DPX mounting medium (Innogenex, San Ramon, CA).

### Quantification of amyloid load by image analysis

Briefly, sagittal slices of all animal groups (n = 5-8 per group) were examined under a microscope (DMI6000B, Leica, Buffalo Grove, IL, USA) and image analyses quantification was performed using the ImagePro software (Rockville, MD, USA). 4G8 immunohistochemistry and ThS staining were quantified in every tenth sections, in a total of 5 sections. All 4G8 and ThS reactive plaques (parenchymal and vascular, induced and naturally occurring) were included in the analyses. Burden was defined as the area of the brain labeled per total area analyzed (data analyses in cortex included all cortical areas). Burden quantification was performed by an investigator blinded to the experimental groups.

### Statistical analysis

Data were expressed as means ± standard error (SEM). Skewness/kurtosis statistic test was used to confirming normal distribution of the data. In all the groups, one-way analysis of variance (ANOVA) followed by a Tukey's multiple comparison analysis was used to determine differences among the groups. The values are expressed as means ± SEM. Data was analyzed using the Graph Pad prism software, version 5.0. *p < 0.05, **p < 0.01, ***p < 0.001. Statistical differences were considered significant for values of p < 0.05.

## Results

### Aβ pathology in AD, MCI and NDAN donor brains

**Figure 1:**
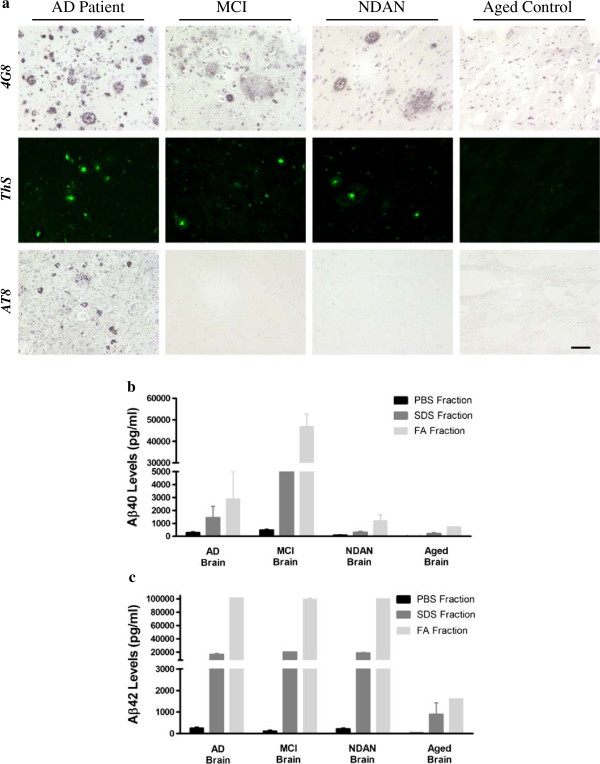
**Aβ deposition patterns in the brain of AD, MCI, NDAN, and Aged control individuals. (a)** The Aβ aggregation profile in the cingulate cortex of a typical AD case was compared to the one present in MCI and NDAN individuals. Slides were stained with an anti-Aβ (4G8) antibody, thioflavin S (ThS) and anti-hyperphosphorylated tau protein (AT8) antibody. Brain slices from an aged non-demented control did not show any AD-associated characteristic (right panels). The scale bar corresponds to 100 μm. **(b and c)** Aβ levels in these brains were evaluated by serial extraction and ELISA as described in Methods. The quantity of Aβ_40_ **(b)** and Aβ_42_ **(c)** was measured using ELISA kits specifically designed to identify these peptides. PBS, Phosphate Buffer Saline; SDS, Sodium Dodecyl Sulfate; FA, Formic Acid. The values were expressed as means ± SEM of the different brains used. Samples were measured in duplicates.

The aggregation and deposition of proteins is one of the main pathological features of AD [3]. To compare the neuropathological changes in MCI and NDAN with the ones observed in a classical AD case, we characterized brain samples from individuals affected by these conditions using different histological techniques. Formalin-fixed samples from the cingulate cortex were stained with thioflavin-S (ThS) to identify amyloid structures, and immunostained using the 4G8 and AT8 antibodies to detect Aβ deposits and hyperphosphorylated-tau, respectively (Figure 1). Extensive accumulation of ThS reactive Aβ deposits was found in the brain of MCI and NDAN individuals (Figure 1a, upper and middle panels), comparable to the one found in the brain from a confirmed AD case. For all three cases, a wide variety of Aβ arrangements, such as parenchymal mature plaques, diffuse aggregates, vascular deposits, and intracellular structures were found. However, established tau pathology was identified only in the AD brain (Figure 1a, lower panels); MCI brains did not display AT8-positive neurons and NDAN samples showed only a couple of reactive neurons in several slices analyzed. As expected, all these pathological characteristics were absent in the brain of a non-demented individual (Figure 1a, right panels).

To further characterize these specimens, we quantified the amount of soluble/insoluble Aβ after serial extraction in phosphate buffered saline (PBS), sodium dodecyl sulfate (SDS), and formic acid (FA) coupled to human-Aβ ELISA. Whereas the levels of insoluble Aβ_40_ (SDS and FA fractions) in the MCI brain were higher compared to the other samples (Figure 1b), the concentration of this molecule in the NDAN preparation showed similar levels to the one observed in a non-demented aged individual, used as control. Interestingly, the concentration of insoluble Aβ_42_ was similar among the AD, MCI and NDAN cases (Figure 1c). Only the brain sample obtained from the aged control showed lower levels of this peptide. The amount of SDS-insoluble/formic acid-soluble Aβ_42_ (representing the most aggregated Aβ species) in AD, MCI and NDAN was ~60-fold higher than in the aged control brain (Figure 1c).

### Characterization of APP_Swe_/PSEN1_∆E9_ mice for studies of induction of Aβ amyloidosis

**Figure 2:**
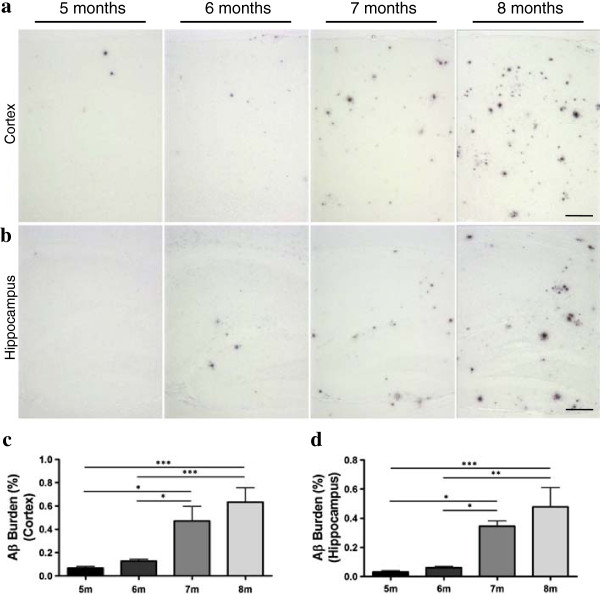
**Time-course of Aβ aggregation in brains of APP**_**Swe**_ **/PS1**_**∆E9**_ **mice.** Brains from 5, 6, 7, and 8 months old animals (n = 5-8) were analyzed by histological staining with an anti-Aβ (4G8) antibody. Representative pictures of Aβ deposition in these mice are shown for the cortical **(a)** and hippocampal areas **(b)**. The burden of amyloid deposits was measured in cortex **(c)** and hippocampus **(d)**. The values were expressed as means ± SEM of the different animals used in each group. The scale bar corresponds to 250 μm.

Several AD-mouse models have been used to study the transmissibility of Aβ misfolding. Most of the transgenic lines previously used involve overexpression of either the wild type or mutant form of the human amyloid precursor protein (APP). Before starting our study, we investigated the appropriate time-frame to assess an exogenous Aβ seeding effect in the double transgenic mouse model used in this study. For this purpose, we followed the time-course of Aβ aggregation and deposition in the brain of APP_Swe_/PSEN1_∆E9_ mice (at 5, 6, 7 and 8 months of age, n = 5-8) by immunohistochemistry. Previous reports suggest that these transgenic animals first start exhibiting amyloid deposits at 4–5 months of age and have extensive plaque deposition at 12 months old [34]. We confirmed these results by observing a time dependent increase of Aβ deposition in both cortex (Figure 2a and 2c) and hippocampus (Figure 2b and 2d). Our data clearly shows that the inflection point in terms of amyloid deposition for this transgenic line is between 6 and 7 months old. The Aβ burden at 7 months old was ~5-fold (cortex) and ~4-fold (hippocampus) higher than in 6 months old mice. Brain samples from male and female subjects showed comparable amounts of Aβ deposits within the same group.

### Injection of brain extracts from MCI and NDAN individuals induces Aβ aggregation in APP_Swe_/PSEN1_∆E9_ mice

**Figure 3:**
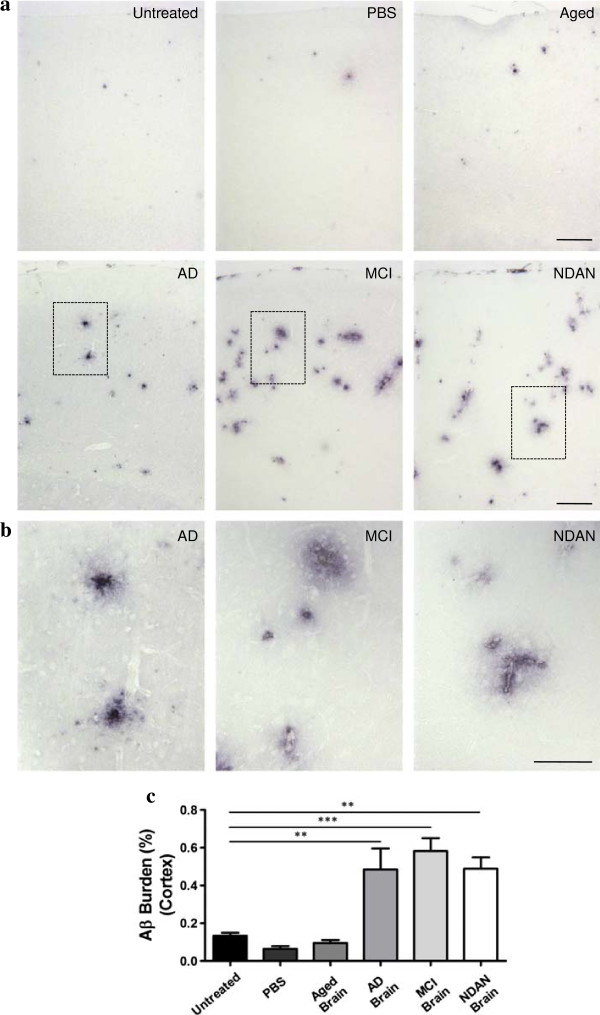
**Injection of brain extracts from MCI and NDAN individuals induce Aβ aggregation in the cortex of AD-transgenic mice.** Serial brain slices of animals inoculated with brain homogenates from different sources (n = 5-8) were analyzed by histological staining using an anti-Aβ (4G8) antibody. **(a)** Representative pictures of the Aβ deposits in different experimental groups and untreated 6 month old animals in cortical area are shown. **(b)** Magnification of the regions labeled in rectangles in panel **(a)** of AD-, MCI-, and NDAN-inoculated animals are shown. The scale bar corresponds to 250 μm in **(a)** and 100 μm in **(b)**. The burden of amyloid deposits was compared among all different control and experimental groups. The 4G8-immunoreative area was measured and divided by the total brain area analyzed (Aβ burden) in the cortex **(c)** of each group.

**Figure 4:**
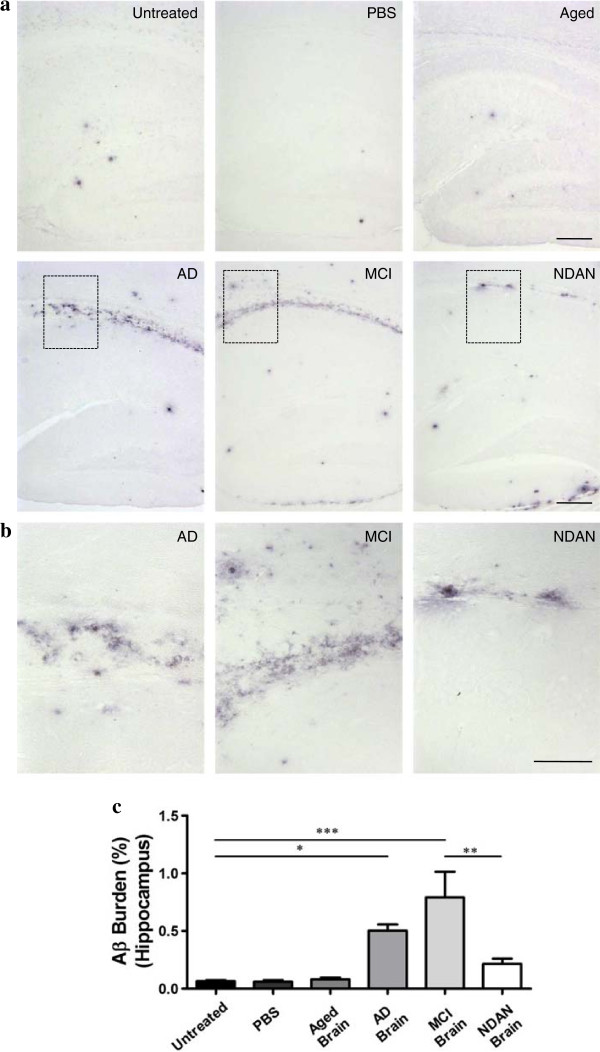
**APP**_**Swe**_ **/PSEN1**_**∆E9**_ **mice inoculated with AD and MCI extracts display higher Aβ deposition in the hippocampus. (a)** Representative pictures of the deposits stained with the 4G8 antibody in the hippocampus of experimental and control animals (n = 5-8). **(b)** Magnification of the labeled regions (corpus callosum) in the pictures shown in **(a)**. The scale bar corresponds to 250 μm in **(a)** and 100 μm in **(b)**. Image analysis was done to quantify the burden of amyloid deposits in the hippocampal area in each group **(c)**. The Aβ deposition was compared among all control and experimental groups.

To test the hypothesis that the sole presence of Aβ aggregates, regardless of the clinical signs, is required to accelerate Aβ deposition in AD-mice, brain extracts from patients affected by MCI or NDAN were intra-cerebrally (i.c.) injected into APP_Swe_/PSEN1_∆E9_ mice at ~1 month old. Additionally, untreated APP_Swe_/PSEN1_∆E9_ mice, as well as mice injected with PBS or brain homogenates obtained from either an AD patient or an aged non-demented individual, were used as controls. All animals were sacrificed ~5 months after inoculation (6 months old), and brains were collected for histopathological analyses. Since seeding effects strongly depends on the incubation periods, the time point selected to analyze the animals was chosen to provide sufficient time to observe an effect and avoid a possible masking due to the endogenous appearance of brain amyloidosis. Brain slices were stained with antibodies against Aβ and the burden of this peptide was measured in hippocampus (injection site). In order to assess if seeding expands from its original placement, we also checked brain cortex, which is an area strongly affected by Aβ lesions in AD patients. As expected, we observed a higher amount of amyloid load in mice injected with the AD extract compared to 6 month old untreated animals and mice injected with PBS or an aged-control brain (Figures 3c and 4c). Strikingly, we found that animals inoculated with the MCI extract had a substantially higher amount of Aβ deposits compared to the control groups (Figures 3 and 4). The Aβ burden was ~5-fold (cortex, Figure 3c) and ~11-fold (hippocampus, Figure 4c) higher compared to that observed in 6 months-old untreated animals. The amyloid load in the cortex of these animals was similar to the one observed in mice inoculated with AD brain (Figure 3c), but the amount in hippocampus was higher (Figure 4c). Interestingly, brains from animals inoculated with the NDAN extract also showed an increased deposition of amyloid aggregates, displaying a high amount of Aβ plaques in the cortical area (Figure 3c) in a similar way as observed for the animals injected with the AD and MCI inocula. The induction of Aβ aggregation by NDAN in the cortex was ~4-fold higher than in 6 months old untreated animals (Figure 3c); however, this treatment did not show any statistically significant difference in the hippocampal area (Figure 4). These results demonstrate that MCI and NDAN brain extracts can accelerate Aβ pathology in a similar manner as AD samples. Nevertheless, the presence of extensive-diffuse accumulation of Aβ reactive deposits in the corpus callosum (Figure 4a) was different from the typical aggregates naturally generated in this model (Figure 2a and 2b).

**Figure 5:**
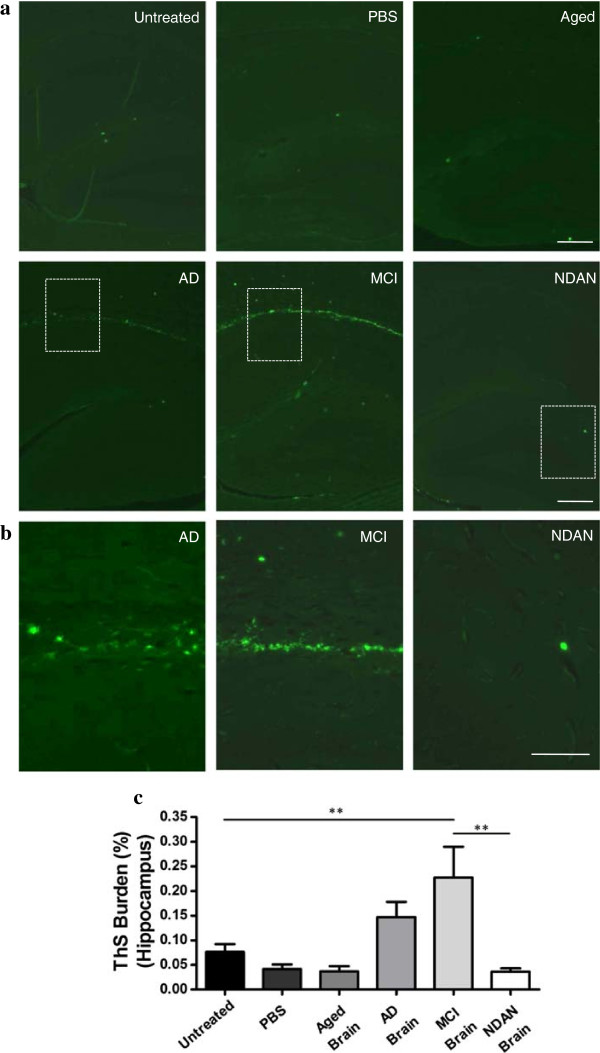
**Injections of brain extracts from AD and MCI patients increase fibrillar Aβ deposition in AD-transgenic animals.** Hippocampal tissue from brain treated and untreated animals (n = 5-8) were stained with ThS and analysed with an epifluorescent microscope. The burden of ThS-positive deposits was compared among all different control and experimental groups. Representative pictures of the deposits in the hippocampus of all groups are shown **(a)**. The scale bar corresponds to 250 μm. **(b)** Magnification of the regions (corpus callosum in AD and MCI, dentate gyrus in NDAN) labeled in rectangles in panel **(a)**. Scale bar correspond to 100 μm. **(c)** The ThS stained area was measured and divided by the total brain area analyzed (ThS burden).

To specifically evaluate the formation of fibrillar amyloid deposits, ThS staining was performed in brains of animals from all experimental and control groups (Figure 5). ThS-reactive deposits were observed mostly in the corpus callosum and fimbria of animals injected with AD or MCI brain extracts (Figure 5a and 5b). Interestingly, the extent of ThS burden in AD and MCI injected animals was lower compared to the Aβ burden measured by antibody binding (Figures 3 and 4), and only reached a statistical significant increase in the hippocampus of animals inoculated with the MCI extract (Figure 5c).

## Discussion

A series of recent and exciting studies have opened the possibility that the pathological accumulation of misfolded protein aggregates implicated in AD and other disorders of protein misfolding could be inducible in a similar manner as prions propagate prion diseases [26–30]. At this time, it is unknown whether the process of transmission of protein misfolding operates only in the spreading of the pathological alterations among cells within an affected individual or can also occur, under certain conditions, to transmit the pathology from individual-to-individual, as described for Creutzfeldt-Jakob disease (CJD). From the experiments performed in animal models, it seems clear that the pathology can be induced by exposure of animals to exogenous tissue homogenates containing aggregates. However, future experiments should address whether the pathology can be induced by more practical and relevant routes of exposure to materials not coming from terminally-sick individuals. In this sense, a putative transmissible origin of Aβ pathology would seem more plausible if Aβ deposition can be also induced by exposure to materials derived from “pre-symptomatic” or “a-symptomatic” cases of AD, since these individuals are more prone to act as donors for tissues and fluids designated for human use. In order to address this issue, we analyzed the amyloid induction capability of samples from individuals affected by MCI (considered as a pre-symptomatic version of AD) and NDAN (which would correspond to an a-symptomatic form of the disease). The particular samples used in this study showed similar amounts of insoluble Aβ in the cingulate cortex and abundant accumulation of amyloid plaques, mostly in cortical and hippocampal areas of the brain. By intra-cerebrally challenging APP_Swe_/PSEN1_∆E9_ transgenic mice with these samples, we demonstrated that the sole presence of Aβ aggregates, regardless of the presence of clinical signs, was sufficient to induce Aβ deposition. Although previous reports have shown a limited induction of Aβ aggregation when brain samples from non-demented individuals were injected to tg2576 [22] and APP23 [23] mice, no reports have shown before a comparison with the pathological induction produced by an AD brain. Since MCI and NDAN samples did not exhibit some of the other alterations typical of AD (e.g. neurofibrillary tangles), our results indirectly support the concept that Aβ aggregates are likely the culprit for the pathological induction. The later conclusion has been strongly supported by the elegant immuno-depletion experiments performed by Jucker and colleagues [23] and the induction of pathology by synthetic Aβ aggregates [35].

Importantly, the seeding effect of AD, MCI and NDAN samples was observed in the cerebral cortex, a brain region anatomically separated from the one used for injection. These results confirm recent findings from us and others showing that Aβ aggregates appear also in the cortex after intrahippocampal injection, suggesting that the pathology can spread to areas other than the injection site [22–24]. This is important, since tissue spreading is one of the main functional features of prion-like proteins [30]. Our results indicate that amyloid seeds coming from MCI and NDAN individuals induced the formation of Aβ deposits with features slightly different than those observed spontaneously in the brain of older untreated transgenic mice. Induced lesions showed a higher proportion of aggregates in the corpus callosum area and structures with lower degree of staining with ThS than those naturally occurring in these animals at advanced age. Importantly, brain homogenate from an aged individual lacking of detectable Aβ structures by histological analyses failed to induce plaque deposition in these mice.

## Conclusions

Our findings demonstrate the prion-like behavior of Aβ aggregates coming from people not formally considered as affected by AD. Hundreds of cases of human-to-human prion transmission have been reported involving blood transfusions, hormone transfer from cadaveric preparations, tissue transplants, and use of contaminated surgical instruments [36]. Importantly, in all these cases, the transmission was from people at the pre-symptomatic stage of the prion disease. Due to their lack of clinical symptoms, MCI and NDAN affected individuals are currently not considered sick, and as in pre-symptomatic CJD transmission, they might be the ones with the higher potential to transmit misfolded seeds to healthy population. Although a recent epidemiological study showed that individuals receiving human-derived growth hormone from future or current AD patients were not at higher risk of developing dementia [37], additional studies directed to confirm/discard the possibility of inter-individual transmission of amyloid pathology in humans are necessary.
